# Pancreatic Polypeptide-Secreting Tumour of the Proximal Pancreas (PPoma)—Ultra Rare Pancreatic Tumour: Clinically Malign, Histologically Benign

**DOI:** 10.3390/medicina55090523

**Published:** 2019-08-23

**Authors:** Ivan Ilić, Vuka Katić, Pavle Randjelović, Nikola Stojanović, Aleksandra Antovic, Ratko Ilić

**Affiliations:** 1 Medical Faculty of Niš, University of Niš, 18000 Niš, Serbia; 2 Policlinic Human, 18000 Niš, Serbia

**Keywords:** pancreatic polypeptide, neuroendocrine tumours, pancreatic neoplasms, PPoma

## Abstract

*Background and objectives:* Here we report a rare case of a pancreatic polypeptide-secreting tumour (PPoma) discovered by accident during an autopsy. These PPomas occur in less than 2% of all pancreatic neoplasms and are almost exclusively silent, i.e., they are non-functional. Symptoms arising from PPoma are due to its compression of surrounding tissue. *Materials and methods:* The autopsy was performed on a 68-year-old male diagnosed with multiple endocrine neoplasm type 1 (MEN1) due to the patient’s sudden death. *Results:* A solitary, densely fibrotic, pink-brown tumour, 18 mm in size tumorous mass, was localised in the head of the pancreas. Microscopically, the tumour had a glandular structure with a tubuloacinar arrangement of the cells. Immunohistochemically, we detected strong PP (pancreatic polypeptide) intracytoplasmic activity and negative glucagon activity. The PPoma was located in the head of the pancreas, likely resulting in the obstruction of the main pancreatic and common bile duct. *Conclusions:* To the best of our knowledge, this is the first report suggesting the association of PPomas with MEN1. Also, the PPoma could be the cause of acute hemorrhagic pancreatitis due to its location.

## 1. Introduction

Pancreatic polypeptide (PP) is a 36 amino acid peptide and 93% of this protein is produced by the F cells of the pancreatic islets [1,2,3]. These F cells (called PP cells as well) can occur scattered through the exocrine parenchyma and can occasionally be found in the pancreatic duct epithelium [4]. Up to now, no real actions of PP have been identified, although earlier suggestions were that it might control gastrointestinal motility and appetite, and function as a safety hormone [3,4,5,6]. Pancreatic neuroendocrine tumours (NETs) can be either non-functional or functional [5,6,7], and this classification is based on the specific pancreatic hormone production from the majority of the neoplastic cells [3,4,5,6,7]. Pancreatic NETs are uncommon and may occur at any age, with the peak of incidence between 30 and 60 years [1,2,3,4,5], where both sexes are equally affected [5,6,7,8].

Pancreatic NETs can be located anywhere in the pancreas. Two-thirds of surgically removed non-functional pancreatic NETs are found in the head of the pancreas [6,9]. This occurrence of a PPoma (pancreatic polypeptide-secreting tumour) is related to the density of F cells, which are mainly found in the islets located in the pancreatic head [10]. These lesions may induce either non-specific abdominal pain or symptoms suggestive of the obstruction of the common bile ducts, or pancreatic ducts [9,10,11,12,13]. The PPoma may occur sporadically or be associated with multiple endocrine neoplasms type 1 (MEN1), and be a part of carcinoid syndrome and hypercalcaemia [2,3,4,5,6].

## 2. Materials and Methods

An autopsy was performed on a 68-year-old male (L.R.) who previously had a variety of symptoms: gout, hypertension, chronic renal failure and a blood sugar level of 230 mg/100 mL. He was previously diagnosed with MEN1, which included massive parathyroid gland chief cell hyperplasia, a functional pituitary ACTH tumour and functional cortical adrenal adenoma. Due to the sudden death of the patient, an autopsy was recommended. Apart from the previous diagnoses, a tumorous mass was clearly visible in the pancreatic tissue. The study was conducted in accordance with the Declaration of Helsinki, and the protocol was approved by the Ethics Committee of the Clinical Centre Nis (12739/ 13 May 2017).

Immunohistochemical staining was performed for the characteristic areas of the tumour, using microscopically selected samples (regions), based on standard Hematoxylin and eosin (H&E) staining. The tissue from the paraffin moulds was cut into 4 µm thick sections and placed on super frost glass slides. Antigen retrieval of the deparaffinised and rehydrated samples was done in a microwave for 20 min in a citric acid buffer (pH 6.0). After cooling to room temperature (rt), the blockage of endogenous peroxidase was performed using 3% hydrogen peroxide. After sample washing (PBS, pH 7.4), primary Pancreatic Polypeptide (Polyclonal Rabbit Anti-Human); Code A0619 and primary Glucagon (Polyclonal Rabbit Anti-Human); Code A0565 (DAKO, Glostrup Denmark) antibodies were applied for 40 min at rt in a moist chamber. Visualisation was achieved by the incubation of the slides with DAKO PAP (Peroxidase Anti-Peroxidase) complexes (Code Z0113, DAKO, Glostrup Denmark) (diluted 1:100) and diaminobenzidine (DAB) or 3-amino-9-ethylcarbazole (AEC) Substrate Chromogen, followed by washing and counterstaining with Mayer’s haematoxylin.

## 3. Results

### 3.1. Macroscopic Findings

**Figure 1:**
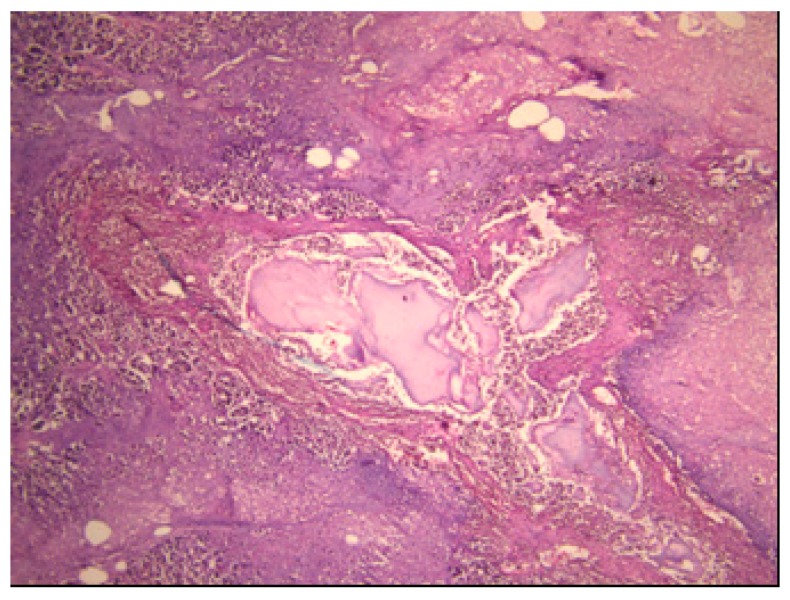
Acute haemorrhagic pancreatitis (H&E (Hematoxylin and eosin) staining; ×40).

A well-demarcated, solitary, densely fibrotic, pink-brown tumour, 18 mm in size, was discovered in the head of the pancreas. The surrounding tissue showed pancreatic steatonecrosis, haemorrhage and multicentric white hard fields of calcification, which were confirmed histologically (Figure 1).

### 3.2. Histopathological Findings

**Figure 2:**
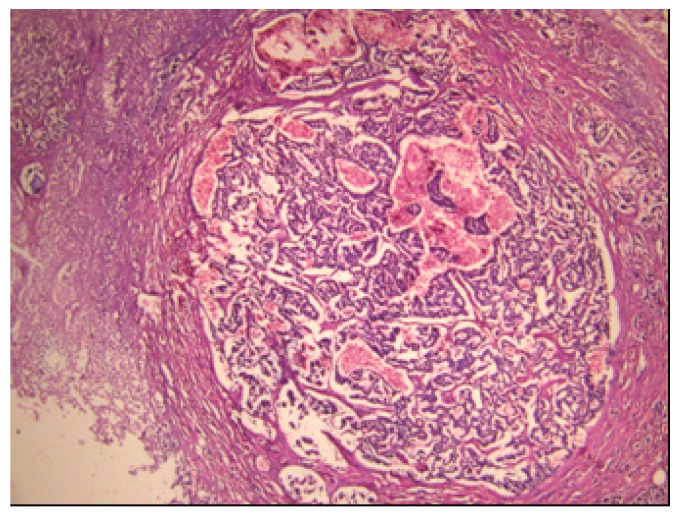
PPoma (pancreatic polypeptide-secreting tumour) with tick capsule: histological pattern (H&E staining; ×40).

**Figure 3:**
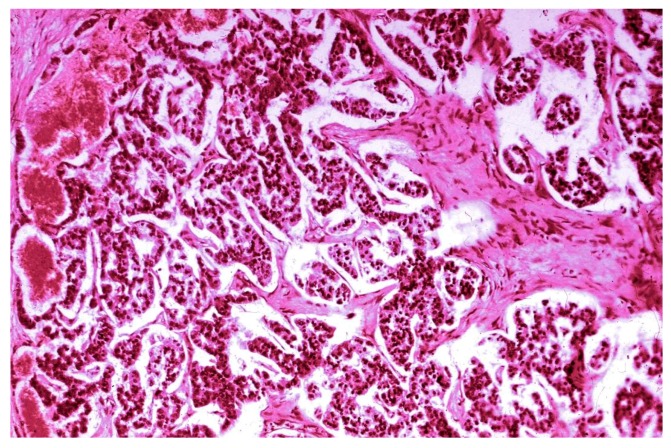
PPoma (pancreatic polypeptide-secreting tumour)—papillary and glandular structure (H&E staining; ×100).

**Figure 4:**
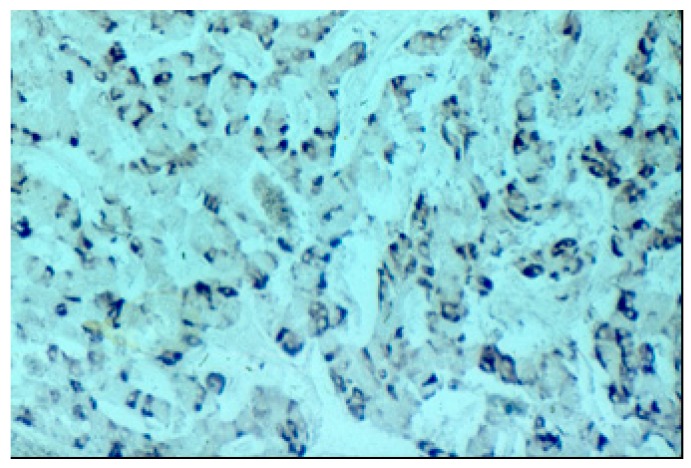
Positive PP (pancreatic polypeptide) staining (PAP (Peroxidase Anti-Peroxidase) ×200 magnification).

**Figure 5:**
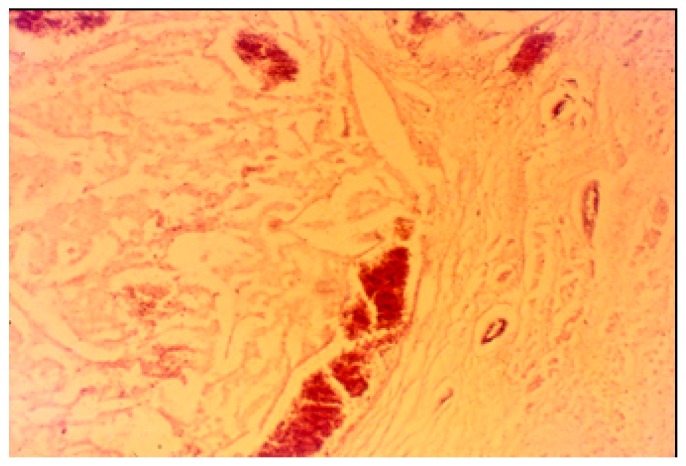
Negative pancreatic glucagon staining (PAP ×200 magnification).

The pancreatic NET was well-differentiated, showing trabecular, glandular, nesting and tubuloacinar arrangements of cells (Figure 2). The cells were relatively uniform, showing finely granular eosinophilic cytoplasm and a centrally located round-oval nucleus (Figure 3). The chromatin pattern was coarsely clumped (“salt and pepper”). In some places, vacuolated, lipid-rich cells were also observed. The final PPoma diagnosis was confirmed immunohistochemically by both positive diffuse and strong PP intracytoplasmic activity (Figure 4). In addition, due to the absence of glucagon activity in this tumour (Figure 5), we have excluded more frequent pancreatic NET-glucagonoma, which is also associated with hyperglycaemia.

## 4. Discussion

Pancreatic neuroendocrine tumours arise from the cells of the endocrine and nervous system within the pancreas and represent one-third of all gastroenteropancreatic neuroendocrine tumours [6,10]. Less frequent aggressive pancreatic NETs have traditionally been termed “islet cell carcinoma” [1,2,3,5,6,7]. Pancreatic NETs are uncommon and represent 1%–2% of all pancreatic neoplasms [5,6,7,8,9,10]. Two-thirds of surgically resected pancreatic NETs are found in the head of the pancreas, which agrees with the autopsy finding presented here. The tumours were removed from patients with back pain and jaundice owing to the obstruction of the common bile ducts [9,10,11,12,13].

Also, PPomas are frequently found to be associated with diabetes mellitus [10], which was the case here. These connections are not fully understood and might appear confusing since PP can directly cause a slight increase in basal plasma insulin concentration. The effects of PP on insulin have been noticed in patients with a resected pancreas or those suffering from chronic pancreatitis where after the PP infusion was administered a reverse in hepatic insulin resistance was observed.

Among the functional neoplasms, insulinomas are usually smaller than glucagonomas, somatostatinomas, gastrinomas or vipomas [11,12,13]. Non-functional NETs are generally >2 cm in diameter, with ill-defined borders, often showing areas of necrosis and haemorrhage [5,10]. Clinically non-functional NETs with immunolabeling for PP in the majority of cells have been designated as “PPomas” [6,9,10]. The secretory granules containing PP are mainly smaller than those of other islet hormones (e.g., glucagon and insulin). Also, these granules are electron dense and appear solid with the dense core extending out to the granule membrane, while on the other hand beta cell granules have a lucent halo around a dense core [4]. Based on the histological pattern of a neoplasm alone, without the determination of its functional state (type of hormone production), a definite diagnosis cannot be reached.

## 5. Conclusions

To the best of our knowledge, this is the first report suggesting the association of PPoma with MEN1. The histological pattern of PPoma is characterised by trabecular, tubular or pseudorosette cell arrangements. The cells are relatively uniform with finely granular eosinophilic cytoplasm and centrally located round to oval nuclei. Based on the strong citoplasmatic PP reactivity and negative glucagon activity, we have confirmed the PPoma diagnosis. The acute hemorrhagic pancreatitis could be attributed to the location of the PPoma and the resultant obstruction of the pancreatic and biliary ducts.
